# The efficacy and safety of general anesthesia vs. conscious sedation for endovascular treatment in patients with acute ischemic stroke: a systematic review and meta-analysis

**DOI:** 10.3389/fneur.2023.1291730

**Published:** 2023-11-17

**Authors:** Jiashuo Zhao, Xin Tan, Xin Wu, Jiaxuan Li, Shixin Wang, Ruisi Qu, Tianchen Chu, Zhouqing Chen, Jiangang Liu, Zhong Wang

**Affiliations:** ^1^Brain and Nerve Research Laboratory, Department of Neurosurgery, The First Affiliated Hospital of Soochow University, Suzhou, Jiangsu, China; ^2^Department of Neurology, The Affiliated Suzhou Hospital of Nanjing Medical University, Suzhou Municipal Hospital, Suzhou, Jiangsu, China

**Keywords:** acute ischemic stroke, endovascular thrombectomy, general anesthesia, conscious sedation, systematic review, meta-analysis

## Abstract

**Background:**

Endovascular thrombectomy (EVT) is an important treatment for patients with acute ischemic stroke (AIS). A number of studies have suggested that anesthesia type (conscious sedation vs. general anesthesia) during intra-arterial treatment for acute ischemic stroke has implications for patient outcomes.

**Methods:**

PubMed, EMBASE, Cochrane Library and clinicaltrials.gov were searched for randomized controlled trials (RCTs) that were performed to evaluate general anesthesia (GA) and conscious sedation (CS) up to May 30, 2023. Review Manager 5.3 software was used to assess the data. The risk ratio (RR) and mean difference (MD) were analyzed and calculated with a fixed effect model.

**Results:**

We pooled 930 patients from seven RCTs. We conducted a meta-analysis comparing the outcomes of GA and CS in the included trials. The rate of functional independence in the GA group was higher than that in the CS group (RR: 1.17, 95% CI: 1.00–1.35; *P* = 0.04; *I*^2^ = 16%). The GA group had a higher successful recanalization rate than the CS group (RR: 1.15, 95% CI: 1.08–1.22; *P* < 0.0001; *I*^2^ = 26%). The GA group had a higher pneumonia rate than the CS group (RR: 1.69, 95% CI: 1.22–2.34; *P* = 0.002; *I*^2^ = 26%). In addition, there was no significant difference between GA and CS with respect to the National Institutes of Health Stroke Scale (NIHSS) score at 24 h (*P* = 0.62), Modified Rankin Scale (mRS) score at 90 days (*P* = 0.25), intracerebral hemorrhage (*P* = 0.54), and mortality at 3 months (*P* = 0.61).

**Conclusion:**

GA demonstrated superiority over CS in achieving successful recanalization and functional independence at 3 months when performing EVT in AIS patients. However, it was also associated with a higher risk of pneumonia. Further studies, particularly those with long-term follow-ups, are necessary to identify precise strategies for selecting the appropriate anesthetic modality in EVT patients.

**Systematic review registration:**

INPLASY202370116.

## 1 Introduction

According to statistics, strokes cause 5.5 million deaths and 116.4 million disabilities annually, making them the third leading risk factor for death globally (1). Acute ischemic stroke (AIS) is the most common type of stroke, accounting for 70% of all strokes (2). AIS is a common and serious neurovascular disorder, that is typically caused by the occlusion or blockage of cerebral arteries supplying blood to the brain. This condition manifests suddenly and is often accompanied by symptoms such as facial paralysis, limb weakness, language difficulties, and visual impairments (3). The acute phase of AIS, which represents the early stage of cerebral ischemia, is the most critical treatment window. Early intervention during this phase can significantly reduce brain damage and improve patient outcomes (4). In the case of early diagnosis of acute ischemic stroke (within 4.5 h of the onset of stroke symptoms), some patients may qualify for intravenous hemolytic clot therapy called recombinant tissue plasminogen activator (Alteplase) (5). The recent research has indicated that, with the advancements in imaging technology, the early treatment window for AIS patients has successfully and safely expanded (6). Even in acute stroke patients with an unknown time of onset, the intravenous administration of alteplase has shown favorable therapeutic outcomes (7). In addition, the American Heart Association (AHA)/American Stroke Association (ASA) guidelines endorsed the use of tenecteplase as a viable choice for thrombolysis in stroke patients within 4.5 h from their last known well time (8). AIS caused by blockages in blood vessels in the brain can be treated by mechanical removal of blood clots (mechanical thrombolysis) from blood vessels within 24 h after stroke symptoms appear (9, 10). Endovascular therapy (EVT) is an interventional treatment method aimed at rapidly restoring impaired cerebral blood supply, thus salvaging brain tissue and function in patients (11). EVT has become the standard of care for acute anterior circulating ischemic stroke and is recommended by the U.S. and European guidelines (12, 13).

Evidence from acute circulatory stroke studies suggests that there are a number of factors affecting the prognosis of patients with acute large vessel occlusion, among which anesthesia and perioperative management may be important (14). At present there are two main methods of anesthesia: general anesthesia (GA) and conscious sedation (CS). CS is associated with potential benefits, including reduced manpower and time requirements, lower costs, fewer hemodynamic fluctuations, and the ability to assess neurological function during the procedure (15). CS can shorten the recanalization time, lower the risk of hypotension or hemodynamic compromise, and be convenient for neurological status monitoring. However, it may draw some concerns such as a longer procedural time due to the movement of the patients, more exposure to radiation, and a lack of airway control (16). In contrast, the advantages of GA encompass airway protection, pain management, patient immobility, and improved radiographic imaging. GA also offers the advantages of ensuring strict immobility, providing airway protection, and avoiding the need for emergency intubation in the event of severe procedural complications (17–19). Whereas, during the induction and recovery phases of GA, there are often significant hemodynamic changes that could potentially exacerbate ischemic injuries (20). In short, the GA combined with intubation may be related to pain, reduced mobility, and a reduced risk of aspiration. CS may be associated with less surgical time, hemodynamic instability, and a lower risk of ventilation-related issues. A previous study showed that GA had lower rates of good functional outcomes and successful angiographic outcomes, as well as higher rates of death, than CS (21). Brinjikji et al. (19) found that when patients with AIS received intraarterial therapy, GA may have worse outcomes than CS. Schonenberger et al.'s (17) study showed no significant improvement in the neurological state of patients undergoing thrombolysis during 24 h of awake sedation compared to GA in patients with anterior circulation acute ischemic stroke. Similarly, Maurice et al.'s (22) study found that functional outcomes three months after endovascular treatment of stroke were similar to those of GA and CS. However, a recent meta-analysis of three randomized clinical trials found that neurological scores in GA EVT patients showed a 14% higher percentage of patients with a good prognosis compared to CS (23). Previous RCTs have been inconclusive about the choice of GA and CS in EVT treatment, but previous studies have focused on anterior circulation stroke (17, 22, 24–27). Liang et al. (28) showed that GA outperformed CS in terms of functional recovery and recanalization success.

The objective of this systematic review and meta-analysis was to examine the outcomes of included RCTs and investigate the effects of GA and CS on the efficacy and safety of AIS patients undergoing EVT, so as to provide new clinical evidences for the selection of anesthesia methods for EVT in AIS patients.

## 2 Methods

### 2.1 Study protocol

Before the project started, we drafted a research protocol following the Cochrane Collaboration format (29). This review was registered with the INPLASY—International Platform of Registered Systematic Review and Meta-analysis Protocols (INPLASY202370116) on July 30, 2023.

### 2.2 Eligibility criteria

We set the inclusion criteria as follows: (1) study type: RCT; (2) language restriction: only available in English; (3) participants: over 18 years of age; acute ischemic stroke (anterior circulation and posterior circulation); artery occlusion confirmed by computed tomographic angiography (CTA), magnetic resonance angiography (MRA), or digital subtraction angiography (DSA); and receiving EVT for artery occlusion; (4) Intervention: GA and CS; GA: procedure in which patients are induced into an unconscious state through various medications; airway protection will also be used, such as tracheal intubation. CS: depression of consciousness using administration of systemic medication; no need for airway protection. (5) Efficacy outcomes: functional independence, defined as the modified Rankin Scale (mRS) 0–2 at 3 months; successful recanalization rate (mTICI 2b-3); National Institutes of Health Stroke Scale (NIHSS) score after 24 h, and mRS score after 90 days. Safety outcomes included mortality after 3 months, and medical complications, such as pneumonia and symptomatic intracerebral hemorrhage (SICH). The definition of symptomatic intracranial hemorrhage is the presence of intracranial hemorrhage as confirmed by imaging, along with an associated NIHSS score of ≥1 within 7 days following the intervention. The included RCTs were not required to supply all the outcomes mentioned above.

We set the exclusion criteria as follows: no report about the aforementioned outcomes or impossibility of extracting the exact number of complications separately from GA and CS, unsuitable study types such as observational studies, case series with sample sizes < 10, case reports, and full texts that, were unavailable.

### 2.3 Search strategy

Two independent investigators (XW and XT) systematically searched Clinicaltrials.gov and three main databases including PubMed, EMBASE, and Cochrane Library to identify relevant studies published until May 30, 2023. The following search strategy was used: [General anesthesia/Conscious sedation (Title/Abstract)] AND [Ischemic Stroke disorder (Title/Abstract)] for PubMed; “General anesthesia/Conscious sedation”/exp AND “Ischemic Stroke disorder”/exp for EMBASE; “General anesthesia/Conscious sedation” in Title Abstract Keyword AND “Ischemic Stroke disorder” in Title Abstract Keyword for Cochrane Library; “General anesthesia/Conscious sedation | Ischemic Stroke disorder” for Clinicaltrials.gov. Additionally, the reference lists of RCTs, relevant systematic reviews and meta-analyses were also screened independently and manually to ensure a more comprehensive search.

### 2.4 Study selection and data collection

According to the eligibility criteria listed above, two reviewers (XW and XT) independently evaluated all study records from the three electronic databases and the reference lists of RCTs and relevant systematic reviews or meta-analyses. Duplicates and research articles that only provided abstracts were excluded. A third reviewer (JSZ) who did not participate in the process of data collection made the final decision regarding the disputed data when disagreements emerged between the two reviewers. After meticulous selection and evaluation, all data from the included RCTs were extracted as follows: the basic information and outcome events included for each trial (Table 1), the inclusion and exclusion criteria, the study design, and all efficacy and safety outcomes are shown in the online Supplementary material (Table 2).

**Table 1:** Characteristics of the included randomized controlled trials.

**Study**	**Countries**	**Centers**	**Outcome events**	**Treatment group (no. of participants)**	**Male (%)**	**Mean age ±SD (years)**	**Mean NIHSS ±SD baseline (score)**	**Occlusion**	**Premorbid mRS 0–2 (no. of participants %)**
• Schonenberger et al. (17) (NCT02126085) • (SIESTA)	Germany	Single	a, c, f, g, h	• GA 73 • CS 77	• 65.8 • 54.5	• 71.8 ± 12.9 • 71.2 ± 14.7	• 16.8 ± 3.9 • 17.2 ± 3.7	Anterior circulation	• 64 (87.6) 71 (92.2)
• Lowhagen Henden et al. (24) • (NCT01872884) • (AnStroke)	Sweden	Single	d, h	• GA 45 • CS 45	• 58.0 • 51.0	• 72.6 ± 11.5 • 73.4 ± 12.3	• 19.5 ± 5.7 • 17.2 ± 5.0	Anterior circulation	• 44 (98) 44 (98)
• Simonsen et al. (25) • (NCT02317237) • (GOLIATH)	Demark	Single	a, e, g	• GA 65 • CS 63	• 55.4 • 47.6	• 71.0 ± 10.0 • 71.8 ± 12.8	• 17.3 ± 6.1 • 17.7 ± 4.6	Anterior circulation	• 63 (96.9) 63 (100)
• Ren et al. (27) • (ChiCTR-IPR-16008494)	China	Single	a, b, e, g, h	• GA 48 • CS 42	• 54.2 • 57.1	• 69.21 ± 5.78 • 69.19 ± 6.46	• 13.6 ± 3.8 • 13.6 ± 3.8	Anterior circulation	• 48 (100) 42 (100)
• Sun et al. (26) • (NCT02677415) • (CANVAS)	China	Single	a, f, g, h	• GA 20 • CS 20	• 65.0 • 65.0	• 67.0 ± 16.0 • 59.3 ± 22.3	• 14.4 ± 5.6 • 13.0 ± 6.4	Anterior circulation	• 20 (100) 20 (100)
• Maurice et al. (22) • (NCT02822144) • (GASS)	French	Multicenter	b, g	• GA 169 • CS 176	• 53.0 • 56.0	• 70.8 ± 13.0 • 72.6 ± 12.3	• 16.0 ± 6.0 • 16.0 ± 5.0	Anterior circulation	• NR
• Liang et al. (28) • (NCT03317535) • (CANVAS II)	China	Multicenter	a, b, f, g, h	• GA 43 • CS 44	• 76.7 • 86.4	• 64.0 ± 11.0 • 60.0 ± 13.0	• 16.4 ± 6.9 • 15.0 ± 4.6	Posterior circulation	• NR

NIHSS, National Institutes of Health Stroke Scale; mRS, modified Rankin Score; NR, not reported; mTICI, modified Thrombolysis in Cerebral Infarction; CS, conscious sedation; GA, general anesthesia.

a, mRS scores at 3 months; b, mRS ≤ 2 at 3 months; c, change in NIHSS score 24 h after intervention; d, difference in mRS scores at 3 months; e, infarct growth 48–72 h after intervention; f, in hospital and 3-month mortality; g, reperfusion rate (mTICI 2b-3); h, pneumonia.

**Table 2:** Inclusion, exclusion criteria, study design and outcome assessments of the included studies.

**Trials**	**Schonenberger et al. (17) (NCT02126085)**
Inclusion criteria	Patients with the following criteria were included: severe ischemic stroke defined by a National Institutes of Health Stroke Scale (NIHSS) score >10 [range, 0–42 with higher scores indicating more severe neurological deficits (a difference of 4 points was considered to be clinically relevant)], isolated or combined occlusion at any level of the internal carotid artery or the middle cerebral artery, decision for thrombectomy according to the internal protocol for acute recanalizing stroke treatment of the Heidelberg University Hospital and at the discretion of the physician in charge
Exclusion criteria	Patients were excluded from the trial if diagnostic imaging results did not clearly depict site of vessel occlusion; their clinical or imaging findings suggested occlusion of a cerebral vessel that was not an internal carotid artery or a middle cerebral artery, or imaging showed intracerebral hemorrhage; coma at admission [Glasgow Coma Scale (GCS) score < 8 (range, 3–15 points with 3 being the worst and 15 the best, composed of 3 parameters: best eye response, best verbal response, and best motor response)]; severe agitation at admission (making groin and vascular access impossible); loss of airway-protective reflexes of at least absence of gag reflex, insufficient saliva handling, observed aspiration, vomiting, or a combination thereof at admission; obviously or known difficult airway; or known intolerance of certain medications for sedation, analgesia, or both
Study design	This was a single-center, parallel-group, open-label RCT with blinded end point evaluation [PROBE (prospective, randomized, open, blinded end point) design]. In this trial, patients selected for thrombectomy were preliminarily randomized 1:1 (using sealed, opaque envelopes based on a computer-generated list not allowing for sequence guessing) to receive either conscious sedation or general anesthesia, standardized according to institutional treatment protocols
Efficacy outcomes	Change in NIHSS score 24 h after intervention; mRS scores at 3 months
Safety outcomes	Adverse events, serious adverse events and death
**Trials**	**Lowhagen Henden et al**. **(****24****)** **(NCT01872884)**
Inclusion criteria	(1) ≥18 years of age, (2) proven occlusion in anterior cerebral circulation by computed tomographic (CT) angiography and NIHSS score ≥10 (if right-sided occlusion) or ≥14 (if left-sided occlusion), and (3) treatment initiated within 8 h after onset of symptoms
Exclusion criteria	(1) The patient was not eligible for randomization because of anesthesiological concerns (airway, agitation, etc) at the discretion of the attending anesthetist, (2) occlusion of posterior cerebral circulation, (3) intracerebral hemorrhage, (4) neurological recovery or recanalization before or during angiography, and (5) premorbidity modified Rankin Scale (mRS) score ≥4 or other comorbidity contraindicating embolectomy
Study design	All admitted patients were directly transported to the CT laboratory where the neurological examination and the CT examination were performed simultaneously. Patients who were eligible for EVT were then transported directly to the neurointerventional suite. In the absence of contraindications, intravenous thrombolysis was started before EVT in all patients. After informed consent, patients were randomly allocated in blocks to either GA or CS in a 1:1 ratio using sealed non-transparent envelopes
Efficacy outcomes	Difference in mRS scores at 3 months; Composite of death, non-fatal stroke, TIA, or peripheral embolism. The NIHSS score shifts at 24 h, day 3, and hospital discharge, as well as cerebral infarction volume at day 3, ASPECTS at day 3
Safety outcomes	Adverse events, serious adverse events and death
**Trials**	**Simonsen et al**. **(****25****)** **(NCT02317237)**
Inclusion criteria	We included all adult patients (18 years of age or older) who presented with large vessel occlusions in the anterior circulation and in whom groin puncture could be performed within 6 h from symptom on set or when last seen well
Exclusion criteria	We excluded patients who were intubated at presentation or with a Glasgow Coma Scale score (score range: 3–15, with a lower score indicating lower levels of consciousness) lower than 9 as well as those who were not living independently and had a premorbid mRS score (score range: 0–6, with a lower score indicating independent living) of more than 2. Because the primary trial end point was infarct growth, we required a diffusion weighted imaging (DWI) MRI scan to establish a baseline (preEVT) infarct volume. Therefore, patients with a contraindication to MRI were excluded. In addition to the DWI scan, the imaging protocol consisted of a T2^*^–a T2 fluid attenuated inversion recovery—and anangiography sequence. Imaging time was 11 min. Patients with baseline infarcts >70 mL were excluded, given their reduced likelihood for achieving good clinical outcomes. Movement or agitation was not a contraindication for the study
Study design	The GOLIATH trial was an investigator-initiated, single-center prospective, randomized, open-label, blinded end-point (or PROBE) evaluation that enrolled patients from March 12, 2015, to February 2, 2017. Patients were randomized to GA or CS in a 1:1 fashion
Efficacy outcomes	The primary outcome was infarct growth, measured in milliliters. Secondary outcome measures were mRS scores after 90 days, time and blood pressure levels
Safety outcomes	Adverse events, serious adverse events and death
**Trials**	**Ren et al**. **(****27****)** **(ChiCTR-IPR-16008494)**
Inclusion criteria	American Society of Anesthesiologists (ASA) grades I–III; National Institutes of Health Stroke Scale (NIHSS) score < 20; AIS within 6.5 h of symptom onset; age ≥60 years; and intracranial proximal arterial occlusion in the anterior circulation (carotid artery, M1 or M2 segments of the middle cerebral artery, or A1 segment of the anterior cerebral artery) demonstrated by computed tomography angiography, magnetic resonance angiography, or digital subtraction angiography (DSA)
Exclusion criteria	Prestroke modified Rankin Scale (mRS) score > 2; hemorrhage demonstrated by computed tomography (CT); obvious or known difficult airway; cognitive impairment; disturbance of consciousness; hypoxemia (SpO2 < 90%); occlusion in the posterior circulation; or body mass index (BMI) >30 kg/m^2^
Study design	In this single-center study, a computer-generated randomization table was used by an independent anesthesia assistant to allocate patients into two groups: the CS group (*n* = 42) and the GA group (*n* = 48)
Efficacy outcomes	The primary outcome was a favorable neurologic outcome at 90 days [favorable outcome was defined as mRS score 0–2 and unfavorable as mRS score 3–6]. Secondary outcomes included baseline characteristics, intraprocedural hemodynamics (recorded at the following time points: arrival at catheterization laboratory [T0]; before puncture [T1]; after angiography [T2]; 3 min [T3], 6 min [T4], 9 min [T5], 12 min [T6], 15 min [T7], 30 min [T8], and 45 min [T9] during the procedure), successful recanalization, time metrics (time interval from stroke onset to catheterization laboratory, catheterization laboratory to groin puncture, and groin puncture to recanalization), vasopressor use, satisfaction score of the neurointerventionalist, complications (pneumonia, other infections, vessel perforation, vessel dissection, distal thrombus, and symptomatic intracerebral hemorrhage, defined as worsening involving NIHSS score ≥1 within 7 days after hemorrhage), the conversion rate from CS to GA, Alberta Stroke Program Early CT Score (ASPECTS) and NIHSS score
Safety outcomes	Adverse events, serious adverse events and death
**Trials**	**Sun et al**. **(****26****)** **(NCT02677415)**
Inclusion criteria	The patients were screened for eligibility if they were admitted with AIS for emergency EVT. The inclusion criteria included patients with age 18 years or older having stroke because of intracranial occlusion, based on single phase, multiphase or dynamic computer tomography angiogram (CTA) or digital subtraction angiography (DSA), at one or more of the following arteries: internal carotid artery (ICA), middle cerebral artery (MCA) segments (M1, and M2) equivalent affecting at least 50% of MCA territory. Patients were eligible only if stroke occurred no more than 6 h from the onset of symptoms and who were previously functionally independent (mRS 0 to 2)
Exclusion criteria	We excluded patients who were moribund with Glasgow coma scale (GCS) score < 8, requiring tracheal intubation for airway protection and lung ventilation. Patients with intracerebral hemorrhage on brain imaging, severely agitation, having seizures, current NIHSS score < 8 or > 35, or known allergy to specific anesthetics (propofol), or analgesics (sufentanil and remifentail) were excluded from the study
Study design	The CANVAS pilot trial is single-center prospective, randomized, open-label, blinded end-point (PROBE) evaluation and enrolled patients with AIS from Beijing Tiantan Hospital, Capital Medical University between April 2016 and June 2017
Efficacy outcomes	mRS after 90 days; favorable outcomes (mRS 0–2); mRS after 30 days; NIHSS after 24 h; NIHSS after 7 days; Reperfusion rate (mTICI 2b-3); Length of ICU stay; Workflow time in mins (symptom to the door; door to arterial puncture; arterial puncture to reperfusion; symptom to reperfusion)
Safety outcomes	Adverse events, serious adverse events and death
**Trials**	**Maurice et al**. **(****22****)** **(NCT02822144)**
Inclusion criteria	We studied patients older than 18 years who had given written informed consent and who were admitted to a participating center for occlusion of a large vessel in the anterior cerebral circulation, admitted for endovascular therapy,17 and affiliated with a social security system
Exclusion criteria	Non-inclusion criteria included patients who were already intubated and mechanically ventilated before inclusion in the study; had intracerebral hemorrhage associated with the ischemic stroke; were contraindicated for conscious sedation (e.g., Glasgow coma scale < 8; agitation preventing patient from staying still during the procedure; deglutition disorder) or succinylcholine (e.g., hyperkalemia, body mass index >35 kg/m^2^); had known allergies to any of the drugs used for anesthesia or to any of their excipients, uncontrolled hypotension, or life-threatening comorbidity; could not walk; had a previous stroke; were pregnant or breastfeeding; were legally protected adults (e.g., under judicial protection, guardianship, or supervision); or were persons deprived of their liberty]
Study design	This was an investigator-initiated, prospective, multicenter, parallel-group, single-blind, randomized, controlled, superiority trial conducted in four centers in France. Patients underwent randomization in a 1:1 ratio to undergo either general anesthesia or conscious sedation. Randomization was centralized and computer generated, and each patient was given a unique randomization number (patient code)
Efficacy outcomes	The primary outcome was the neurologic outcome assessed by modified Rankin score between 2 and 6 months after the endovascular treatment. Secondary outcomes were time from stroke onset to groin puncture; time from arrival in the stroke center to groin puncture; technical failure of the endovascular treatment (defined as failure of arterial puncture or catheterization); reperfusion results evaluated by the neuroradiologist (good reperfusion corresponded to a modified treatment in cerebral ischemia scale score of 2b or 3); National Institutes of Health Stroke Scale score at day 1 (i.e., day after the endovascular treatment) and day 7 (or the day the patient left the hospital if scheduled before day 7)
Safety outcomes	Adverse events, serious adverse events and death
**Trials**	**Liang et al**. **(****28****)** **(NCT03317535)**
Inclusion criteria	Eligible candidates were patients 18 years and older with acute PCS (basilar artery or vertebral artery) discovered by computed tomography angiography or magnetic resonance angiography whose condition was suitable for recanalization treatment with < 24 h from onset to primary treatment and whose modified Rankin Scale (mRS) score was 2 or lower before the stroke occurred
Exclusion criteria	Exclusion criteria included unclear radiological images for identifying infarction and vessel occlusion, anterior circulation occlusion, intracranial hemorrhage, posterior circulation Acute Stroke Prognosis Early Computed Tomography score < 6, pons-midbrain index score 3 or greater, severe agitation or seizures, loss of airway protective reflexes and/or vomiting on admission, intubation before EVT, unconsciousness, known allergy to anesthetics or analgesics, and refusal to participate on the part of the patient or their legal representative. Before recruitment, patients had to obtain agreement from the neuroradiologist and anesthesiologist that they were suitable for GA or CS
Study design	This is a double-center randomized parallel-group exploratory Choice of Anesthesia for Endovascular Treatment of Acute Ischemic Stroke in Posterior Circulation (CANVAS II) trial. Enrolled participants were randomized in a 1:1 ratio for treatment with GA or CS
Efficacy outcomes	The primary end point was functional independence, defined as an mRS score of 2 or lower at 90 days. Secondary outcomes included changes in NIHSS score from baseline to 30 and 90 days after randomization; modified treatment in cerebral infarction (mTICI) score at baseline and after treatment; conversion rate; all-cause mortality and proportions of complications up to 90 days after randomization; and time-related outcomes, such as treatment time, length of stay in the hospital and intensive care unit, and time from onset to door
Safety outcomes	Adverse events, serious adverse events and death

### 2.5 Risk of bias

The risk of bias plot for individual studies was assessed with Review Manager 5.3 software. The uniform criteria to assess the risk of bias for RCTs of the Cochrane Collaboration were applied, which included: selection bias, performance bias, detection bias, attrition bias, reporting bias, and other potential biases. Each bias criterion was classified as “low,” “high,” or “unclear” after independently judging by the third reviewer.

### 2.6 Statistical analysis

Review Manager 5.3 software was used to assess the data. For the dichotomous outcomes, the risk ratio [relative risk (RR); 95% confidence interval (CI)] was analyzed and calculated with a fixed effect model. Mean difference (MD) was used for continuous outcomes such as the NIHSS score at 24 h and the mRS score at 90 days. Heterogeneity was estimated via the *I*^2^ statistic, which was as follows: *I*^2^ < 30% suggests “low heterogeneity”; *I*^2^ between 30 and 50% means “moderate heterogeneity”; and *I*^2^ > 50% denotes “substantial heterogeneity”. A sensitivity analysis was used to explore the stability of the consolidated results. For all the analyses, two tailed tests were performed and a *P* value < 0.05 was considered statistically significant.

## 3 Results

**Figure 1:**
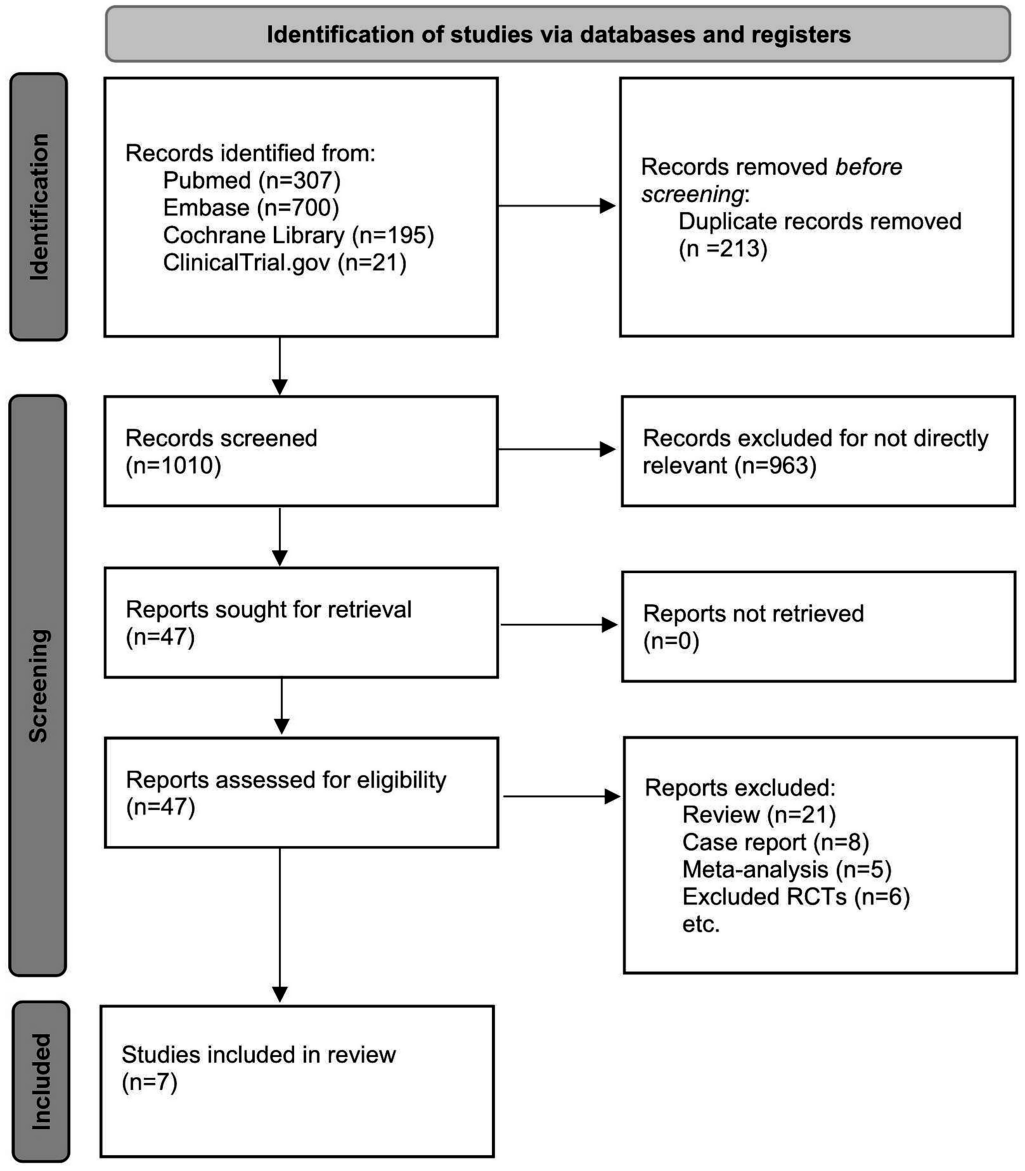
The study search, selection, and inclusion process.

A total of 1,010 titles and abstracts were returned from the search through PubMed, EMBASE, Cochrane Library and Clinicaltrials.gov. After quick of screening the titles and abstracts, a total of 963 articles were excluded due to duplication and irrelevance and 47 full text articles were assessed for eligibility. Among them, another 40 articles were excluded due to the limitation of publication types: six non-randomized clinical trials, eight case reports, five meta-analyses and 21 reviews. The selection process is summarized in the flow diagram (Figure 1). All seven selected RCTs enrolling 930 patients were pooled for the analyses of efficacy and safety outcomes. The main characteristics of the seven included studies are listed in Table 1.

### 3.1 Efficacy outcomes analysis

**Figure 2:**
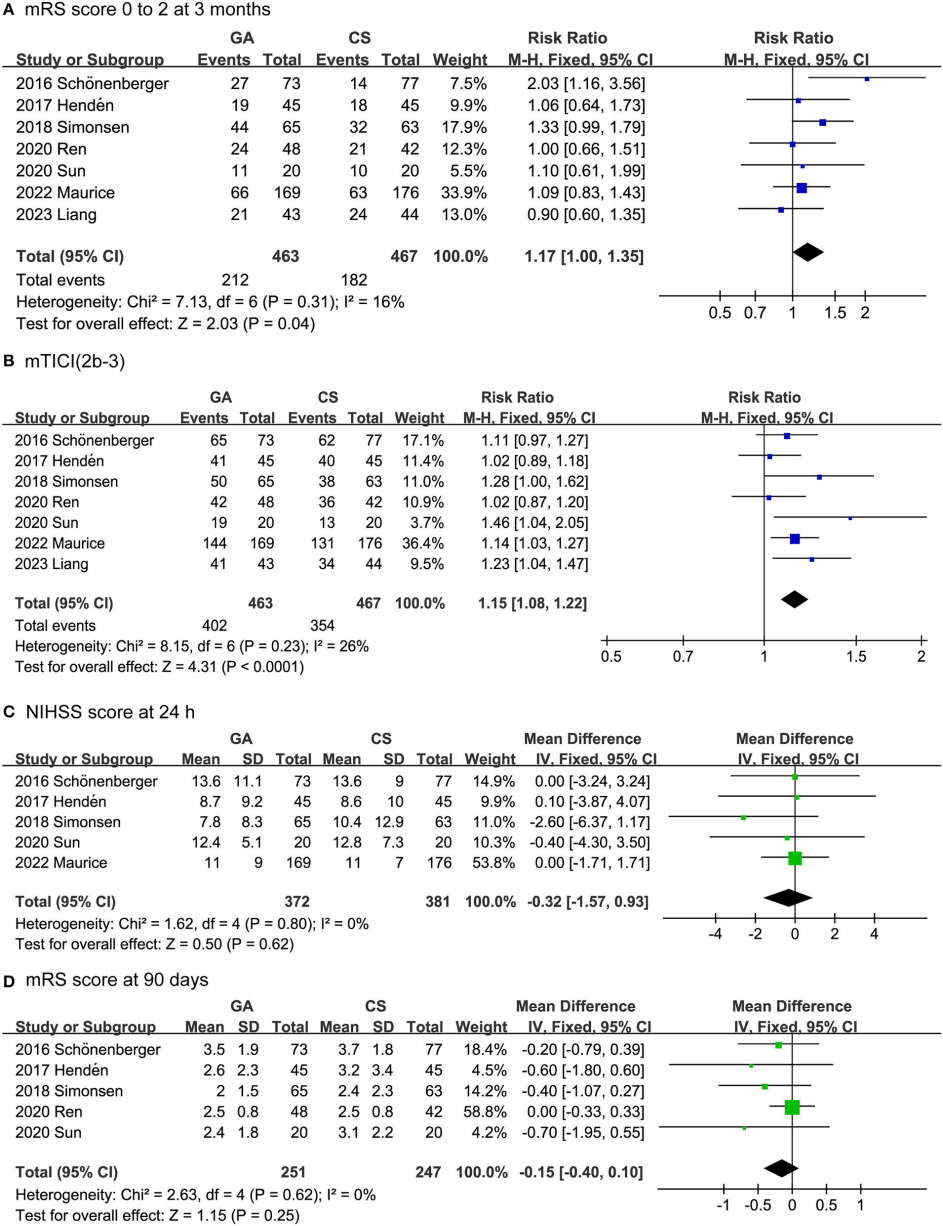
Forest plots for efficacy outcomes. **(A)** mRS score 0 to 2 at 3 months. **(B)** Successful recanalization rate. **(C)** NIHSS score after 24 h. **(D)** mRS score after 90 days.

The efficacy outcomes included the mRS score 0 to 2 at 3 months, successful recanalization rate (mTICI 2b-3) [Successful reperfusion rate (mTICI 2b-3) is a measure of the degree of blood flow restoration in the brain after an ischemic stroke. It is defined as achieving a score of 2b or 3 on the modified Thrombolysis in Cerebral Infarction (mTICI) scale, which ranges from 0 (no perfusion) to 3 (complete perfusion)] (30), NIHSS score after 24 h, and mRS score after 90 days. As shown in Figure 2, the rate of functional independence in the GA group was higher than that in the CS group (RR: 1.17, 95% CI: 1.00–1.35; *P* = 0.04; *I*^2^ = 16%). Likewise, the GA group had a higher successful recanalization rate than the CS group (RR: 1.15, 95% CI: 1.08–1.22; *P* < 0.0001; *I*^2^ = 26%). On the other hand, there was no difference between the GA and CS groups in NIHSS score at 24 h (MD: −0.32, 95% CI: −1.57 to 0.93; *P* = 0.62; *I*^2^ = 0%) or mRS score at 90 days (MD: −0.15, 95% CI: −0.40 to 0.10; *P* = 0.25; *I*^2^ = 0%).

### 3.2 Safety outcomes analysis

**Figure 3:**
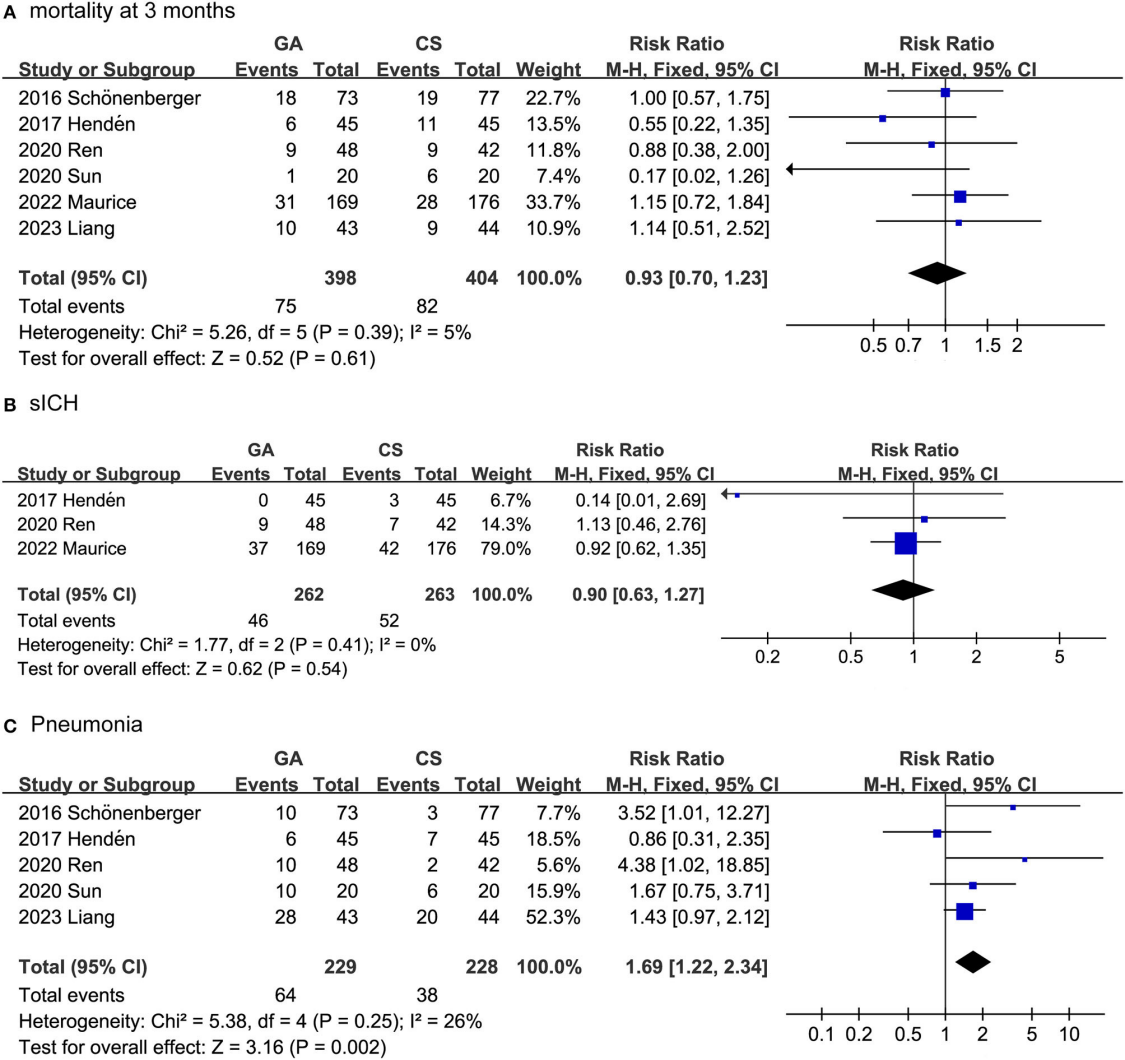
Forest plots for safety outcomes. **(A)** Mortality after 3 months; **(B)** SICH; **(C)** pneumonia.

The safety outcomes included mortality after 3 months, SICH and pneumonia. As shown in Figure 3, the safety outcomes were assessed by adverse events and serious adverse events. In fact, we combined the data collected from the seven trials and surprisingly found that the GA group had a higher pneumonia rate than the CS group (RR: 1.69, 95% CI: 1.22–2.34; *P* = 0.002; *I*^2^ = 26%). For the collected data, there was no difference between the GA and CS groups in SICH (RR: 0.90, 95% CI: 0.63–1.27; *P* = 0.54; *I*^2^ = 0%), or mortality at 3 months (RR: 0.93, 95% CI: 0.70–1.23; *P* = 0.61; *I*^2^ = 5%).

### 3.3 Risk of bias in included studies

**Figure 4:**
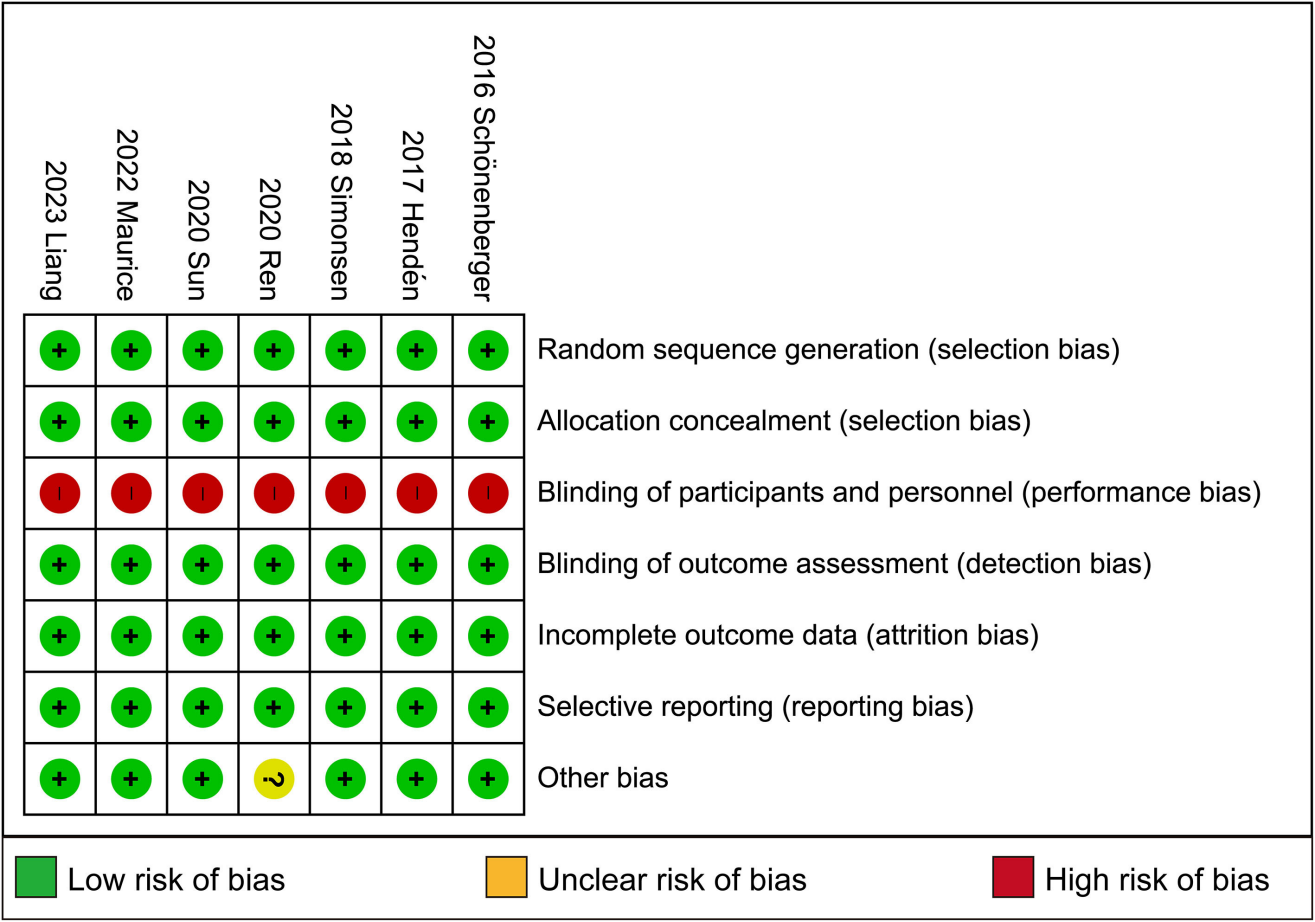
Risk of bias: a summary table for each risk of bias item for each study.

Full details of the risk bias for all enrolled studies are shown in Figure 4. All seven clinical trials showed a low risk of bias in both random sequence generation and allocation concealment. For the blinding of participants and personnel and the blinding of outcome assessment, the risk of bias was high in all seven trials. Apart from these items, an unclear risk of bias was also observed in all RCTs.

## 4 Discussion

Our study systematically reviewed and meta-analyzed data from seven previous RCTs, collecting 930 valid cases. The results showed that, in AIS patients under GA, successful EVT recanalization (87 vs. 76%) and functional independence (46 vs. 39%) were higher than those in the CS group. Based on the outcome indicators we included, the NIHSS score and 90-day mRS scores in the GA and CS groups were not statistically significant. GA, on the other hand, was associated with a higher risk of pneumonia (28 vs. 17%). However, GA mortality at 3 months (19 vs. 20%) and SICH (18 vs. 20%) were similar to CS.

For recent years, EVT has been a therapeutic option for patients with AIS caused by large vessel occlusion (LVO) (31). Previous trials have confirmed its safety and effectiveness in treating anterior circulation LVO strokes (32, 33). The choice of anesthesia for EVT is particularly important. Emphasis must therefore be placed on intraoperative respiratory and circulatory management, in which the choice of anesthesia method plays an important role. Previous studies have focused on anterior circulation, but posterior circulation, due to its low incidence and poor prognosis, has rarely been studied. Recently, Liang et al. (28) reported an RCT comparing the use of GA and CS in the treatment of acute posterior circulation ischemic stroke. Therefore, our study combined anterior and posterior circulation.

According to our results, AIS patients with GA significantly outperformed those with CS at 3 months of functional independence. In Wang et al.'s (34) study, patients treated with GA during EVT were less likely to be functionally independent within 90 days and to have a lower rate of good return than those treated with CS. This is not consistent with our results. Through our study, functional independence was significantly higher in AIS patients under GA than in those under CS (46 vs. 39%) within 3 months. Notably, Wang et al.'s study was based on observational, non-RCTs, and the design of the study can influence the relationship with functional outcomes.

Next, the post-EVT recanalization success rate of AIS patients was one of the main outcome indicators we included. Our study showed a significantly higher recanalization success rate in the GA group than in the CS group (87 vs. 76%). Successful recanalization and functional independence are the main criteria for evaluating EVT efficacy. In addition, successful recanalization was closely related to functional independence 3 months after surgery. Campbell et al. (35) suggest that this may be due to the superior procedural conditions offered by patient immobilization and control of apnea during EVT. In addition, GA advantages, such as monitoring physiological parameters such as oxygenation and hemodynamics, may also contribute to better EVT outcomes. However, Davis et al. found that ischemic stroke patients who received endovascular recanalization experienced higher intraprocedural hypotension under GA than under CS. They suggest that the induction and recovery phases of GA are usually associated with significant hemodynamic changes (hypotension and rapid blood pressure fluctuations), which may exacerbate ischemic injury, leading to lower recanalization success (36). However, our study showed that the GA group had a higher recanalization rate. While GA influences blood pressure fluctuations in EVT patients, it renders them unconscious or unresponsive. This state of unconsciousness promotes improved patient cooperation during surgical procedures, enabling physicians to achieve enhanced visibility and control during the intervention, ultimately leading to an improvement in the recanalization success rate. Therefore, the conclusion that GA may have more potential than CS to promote good outcomes should be cautiously drawn.

On the other hand, we looked at the incidence of pneumonia and found that the incidence in the GA group was higher than that in the CS group (28 vs. 17%). This is consistent with the findings of Wan et al. (37). GA typically involves placing the patient in a deep state of unconsciousness and using mechanical ventilation to support respiration. This may lead to the retention of respiratory secretions and a reduced ability to clear them, thereby increasing the risk of pneumonia. GA can result in postoperative decline in lung function, particularly in the case of longer surgeries (38). This decrease in lung function can potentially raise the risk of patients developing pneumonia. Despite the higher incidence of pneumonia in the GA group compared to the CS group, our study found that even within the CS group, 17% of patients developed pneumonia. Some commonly used CS-related anesthetic drugs, such as neuromuscular blocking agents, may increase the risk of patients developing pneumonia because they can lead to respiratory muscle paralysis and the risk of aspiration (39). Therefore, the incidence of pneumonia cannot be ignored when EVT is administered under either anesthetic. Further research is needed to strengthen perioperative respiratory care, encourage patients to get out of bed as early as possible, especially after GA, promote neurological recovery and avoid pneumonia. Although we have observed risk factors for the development of pneumonia, which can provide a comprehensive assessment for the selection of anesthetic approaches, these finding may lack versatility and require, in particular, support from mature multidisciplinary collaboration between neurointerventionists and anesthesiologists.

The NIHSS score was used to assess the severity of AIS in all participants in the study. Based on the outcome indicators we included, the 24 h NIHSS score and 90-day mRS scores in the GA groups and CS groups were not statistically significant. Movement of a patient's limb during surgery can lead to wire perforation, intracranial bleeding or vascular damage in the form of dissection. GA may reduce limb movement in patients undergoing endovascular therapy, and awake CS patients may experience limb movement during endovascular therapy, which may affect the safety and effectiveness of interventional therapy. However, by analyzing the data included in the study, there was no difference in risk factors for intracranial hemorrhage between the GA group and the CS group.

Finally, according to our study, the conversion rate from CS to GA was 8% in the anterior circulation study and 29.5% in the posterior circulation study. The most common reasons for switching to GA are restlessness and mental changes during surgery. In the posterior circulation study, rapid progression of the disease was accompanied by a rapid decline in consciousness and respiratory circulation parameters, leading to a high CS conversion rate. Previous studies may have underestimated the high conversion rate in CS groups. In other words, patients in the CS group may experience more technical glitches, while those in the GA group may experience more episodes of hypotension and better recanalization.

The current study still has some limitations. There was a low number of studies included. In addition, the choice of different anesthetic drugs and different types of general anesthesia (intravenous, inhaled) may affect the outcome of the trial. Additionally, there were slight variations in the definition of symptomatic cerebral hemorrhage among the studies we included, which had an impact on our data analysis, leading to some degree of heterogeneity. Previous studies have focused on anesthesia options for EVT in patients with acute anterior circulation stroke, with few studies of acute posterior circulation stroke. Compared to anterior circulating stroke, posterior circulation stroke mainly involves the brain stem, which controls many physiological functions essential to life, such as breathing, heart rate and blood pressure (40). Previous studies have analyzed only one type of anterior circulation or posterior circulation, and our study is the first to incorporate both types of circulation, which leads to a degree of heterogeneity. We found that *I*^2^ < 50% for all outcome measures, and subsequently, we performed a sensitivity analysis as shown in Supplementary Figure S1. By excluding studies related to the posterior circulation and reanalyzing the data, This did not significantly impact the pooled results, so we consider our results to be robust. In the future, further high-quality research will be necessary to obtain new clinical evidence regarding anesthesia choices for EVT.

## 5 Conclusion

In this systematic review and meta-analysis, EVT for AIS patients conducted under GA demonstrated a superior recanalization success rate and greater functional independence at the 3-month mark when contrasted with CS. On the other hand, GA is associated with a higher risk of pneumonia. More research is needed in the future, especially those with long-term follow-ups, to identify precision strategies for patients with anesthetic modality selection during EVT.

## Data availability statement

The original contributions presented in the study are included in the article/Supplementary material, further inquiries can be directed to the corresponding authors.

## Author contributions

JZ: Conceptualization, Writing – original draft, Writing – review & editing. XT: Conceptualization, Investigation, Writing – review & editing. XW: Data curation, Writing – review & editing. JLi: Methodology, Writing – review & editing. SW: Supervision, Writing – review & editing. RQ: Project administration, Writing – review & editing. TC: Funding acquisition, Writing – review & editing. ZC: Supervision, Writing – review & editing. JLiu: Funding acquisition, Writing – original draft. ZW: Funding acquisition, Supervision, Writing – original draft.

## Supplementary material

Supplementary Table S1 Search strategy of studies.

Supplementary Figure S1 Sensitivity analysis.
