# Supplementary material for: The efficacy and safety of general anesthesia vs. conscious sedation for endovascular treatment in patients with acute ischemic stroke: a systematic review and meta-analysis
Source: Front Neurol. 2023 Nov 17;14:1291730. doi: 10.3389/fneur.2023.1291730 (PMC10690773; doi:10.3389/fneur.2023.1291730)
Supplement: Supplementary Table S1 — Search strategy of studies. [file Table_1.docx]

**Search Strategy**

Search Date: 30 May 2023

**Pubmed：**

| Search | Query | Results |
| --- | --- | --- |
| #1 | "Ischemic Stroke"[Mesh] | 9382 |
| #2 | ((((((((((((((((((((((Ischemic Strokes[Title/Abstract]) OR (Stroke, Ischemic[Title/Abstract])) OR (Ischaemic Stroke[Title/Abstract])) OR (Ischaemic Strokes[Title/Abstract])) OR (Stroke, Ischaemic[Title/Abstract])) OR (Cryptogenic Ischemic Stroke[Title/Abstract])) OR (Cryptogenic Ischemic Strokes[Title/Abstract])) OR (Ischemic Stroke, Cryptogenic[Title/Abstract])) OR (Stroke, Cryptogenic Ischemic[Title/Abstract])) OR (Cryptogenic Stroke[Title/Abstract])) OR (Cryptogenic Strokes[Title/Abstract])) OR (Stroke, Cryptogenic[Title/Abstract])) OR (Cryptogenic Embolism Stroke[Title/Abstract])) OR (Cryptogenic Embolism Strokes[Title/Abstract])) OR (Embolism Stroke, Cryptogenic[Title/Abstract])) OR (Stroke, Cryptogenic Embolism[Title/Abstract])) OR (Wake-up Stroke[Title/Abstract])) OR (Stroke, Wake-up[Title/Abstract])) OR (Wake up Stroke[Title/Abstract])) OR (Wake-up Strokes[Title/Abstract])) OR (Acute Ischemic Stroke[Title/Abstract])) OR (Acute Ischemic Strokes[Title/Abstract])) OR (Stroke, Acute Ischemic[Title/Abstract]) | 35106 |
| #3 | #1 OR #2 | 39680 |
| #4 | "Anesthesia, General"[Mesh] | 61694 |
| #5 | ((Anesthesias, General[Title/Abstract]) OR (General Anesthesia[Title/Abstract])) OR (General Anesthesias[Title/Abstract]) | 74578 |
| #6 | #4 OR #5 | 112962 |
| #7 | "Conscious Sedation"[Mesh] | 9678 |
| #8 | ((Sedation, Moderate[Title/Abstract]) OR (Moderate Sedation[Title/Abstract])) OR (Sedation, Conscious[Title/Abstract]) | 778 |
| #9 | #7 OR #8 | 10131 |
| #10 | #6 OR #9 | 121272 |
| #11 | #3 AND #10 | 307 |
| #12 | "Randomized controlled trial"[Filter] | 589969 |
| #13 | #11 AND #12 | 22 |

**Embass：**

| Search | Query | Results |
| --- | --- | --- |
| #1 | 'acute ischemic stroke'/exp | 37756 |
| #2 | 'ischemic strokes':ab,ti,kw | 7357 |
| #3 | 'stroke, ischemic':ab,ti,kw | 1398 |
| #4 | 'ischaemic stroke':ab,ti,kw | 14359 |
| #5 | 'ischaemic strokes':ab,ti,kw | 1248 |
| #6 | 'stroke, ischaemic':ab,ti,kw | 262 |
| #7 | 'cryptogenic ischemic stroke':ab,ti,kw | 376 |
| #8 | 'cryptogenic ischemic strokes':ab,ti,kw | 41 |
| #9 | 'ischemic stroke, cryptogenic':ab,ti,kw | 9 |
| #10 | 'stroke, cryptogenic ischemic':ab,ti,kw | 0 |
| #11 | 'cryptogenic stroke':ab,ti,kw | 3635 |
| #12 | 'cryptogenic strokes':ab,ti,kw | 499 |
| #13 | 'stroke, cryptogenic':ab,ti,kw | 28 |
| #14 | 'cryptogenic embolism stroke':ab,ti,kw | 1 |
| #15 | 'cryptogenic embolism strokes':ab,ti,kw | 0 |
| #16 | 'embolism stroke, cryptogenic':ab,ti,kw | 0 |
| #17 | 'stroke, cryptogenic embolism':ab,ti,kw | 0 |
| #18 | 'wake-up stroke':ab,ti,kw | 490 |
| #19 | 'stroke, wake-up':ab,ti,kw | 22 |
| #20 | 'wake up stroke':ab,ti,kw | 490 |
| #21 | 'wake-up strokes':ab,ti,kw | 174 |
| #22 | 'acute ischemic stroke':ab,ti,kw | 36261 |
| #23 | 'acute ischemic strokes':ab,ti,kw | 803 |
| #24 | 'ischemic stroke, acute':ab,ti,kw | 176 |
| #25 | 'stroke, acute ischemic':ab,ti,kw | 50 |
| #26 | #1 OR #2 OR #3 OR #4 OR #5 OR #6 OR #7 OR #8 OR #9 OR #10 OR #11 OR #12 OR #13 OR #14 OR #15 OR #16 OR #17 OR #18 OR #19 OR #20 OR #21 OR #22 OR #23 OR #24 OR #25 | 62926 |
| #27 | 'general anesthesia'/exp | 113301 |
| #28 | 'anesthesias, general':ab,ti,kw | 2 |
| #29 | 'general anesthesia':ab,ti,kw | 64260 |
| #30 | 'general anesthesias':ab,ti,kw | 55 |
| #31 | #27 OR #28 OR #29 OR #30 | 135200 |
| #32 | 'conscious sedation'/exp | 9659 |
| #33 | 'sedation, moderate':ab,ti,kw | 28 |
| #34 | 'moderate sedation':ab,ti,kw | 1379 |
| #35 | 'sedation, conscious':ab,ti,kw | 41 |
| #36 | #32 OR #33 OR #34 OR #35 | 10154 |
| #37 | #31 OR #36 | 142930 |
| #38 | #26 AND #37 | 700 |
| #39 | 'random':ab,ti OR 'control':ab,ti OR 'double-blind':ab,ti | 4489120 |
| #40 | #38 AND #39 | 71 |

**Cochrane:**

| Search | Query | Results |
| --- | --- | --- |
| #1 | MeSH descriptor: [Ischemic Stroke] explode all trees | 732 |
| #2 | (Ischemic Strokes):ti,ab,kw OR (Stroke, Ischemic):ti,ab,kw OR (Ischaemic Stroke):ti,ab,kw OR (Ischaemic Strokes):ti,ab,kw OR (Stroke, Ischaemic):ti,ab,kw | 18016 |
| #3 | (Cryptogenic Ischemic Stroke):ti,ab,kw OR (Cryptogenic Ischemic Strokes):ti,ab,kw OR (Ischemic Stroke, Cryptogenic):ti,ab,kw OR (Stroke, Cryptogenic Ischemic):ti,ab,kw OR (Cryptogenic Stroke):ti,ab,kw | 262 |
| #4 | (Cryptogenic Strokes):ti,ab,kw OR (Stroke, Cryptogenic):ti,ab,kw OR (Cryptogenic Embolism Stroke):ti,ab,kw OR (Cryptogenic Embolism Strokes):ti,ab,kw OR (Embolism Stroke, Cryptogenic):ti,ab,kw | 262 |
| #5 | (Stroke, Cryptogenic Embolism):ti,ab,kw OR (Wake-up Stroke):ti,ab,kw OR (Stroke, Wake-up):ti,ab,kw OR (Wake up Stroke):ti,ab,kw OR (Wake-up Strokes):ti,ab,kw | 392 |
| #6 | (Acute Ischemic Stroke):ti,ab,kw OR (Acute Ischemic Strokes):ti,ab,kw OR (Ischemic Stroke, Acute):ti,ab,kw OR (Stroke, Acute Ischemic):ti,ab,kw | 8895 |
| #7 | #1 OR #2 OR #3 OR #4 OR #5 OR #6 | 18299 |
| #8 | MeSH descriptor: [Anesthesia, General] explode all trees | 7414 |
| #9 | (Anesthesias, General):ti,ab,kw OR (General Anesthesia):ti,ab,kw OR (General Anesthesias):ti,ab,kw | 29656 |
| #10 | #8 OR #9 | 31152 |
| #11 | MeSH descriptor: [Conscious Sedation] explode all trees | 1559 |
| #12 | (Sedation, Moderate):ti,ab,kw OR (Moderate Sedation):ti,ab,kw OR (Sedation, Conscious):ti,ab,kw | 5314 |
| #13 | #11 OR #12 | 5314 |
| #14 | #10 OR #13 | 35625 |
| #15 | #7 AND #14 | 195 |

**Clinicaltrials.gov：**

| Search | Query | Results |
| --- | --- | --- |
| #1 | Status:All studies,condition or disease: Acute Ischemic Stroke and other terms: General Anesthesia, Study type: interventional(Clinical Trial) | 9 |
| #2 | Status:All studies,condition or disease: Acute Ischemic Stroke, and other terms: Conscious Sedation, Study type: interventional(Clinical Trial) | 12 |
