# Supplementary figures and images for: The efficacy and safety of general anesthesia vs. conscious sedation for endovascular treatment in patients with acute ischemic stroke: a systematic review and meta-analysis
Source: Front Neurol. 2023 Nov 17;14:1291730. doi: 10.3389/fneur.2023.1291730 (PMC10690773; doi:10.3389/fneur.2023.1291730)

### A.mRS score 0 to 2 at 3 months

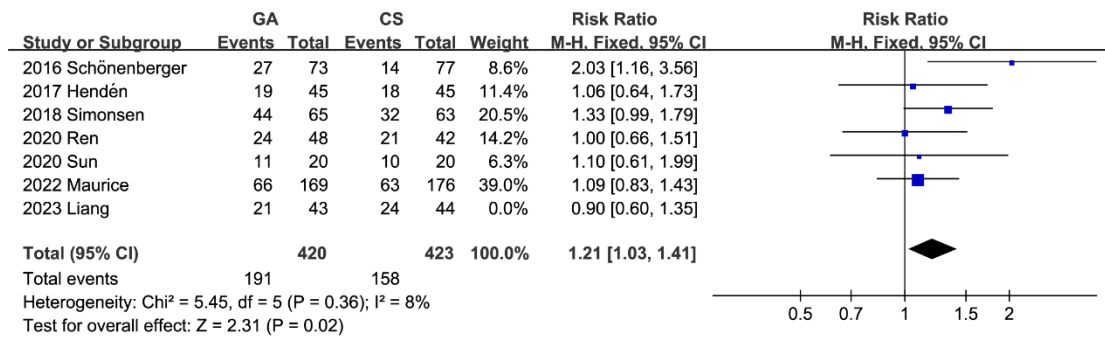

### B.mTICI(2b-3)

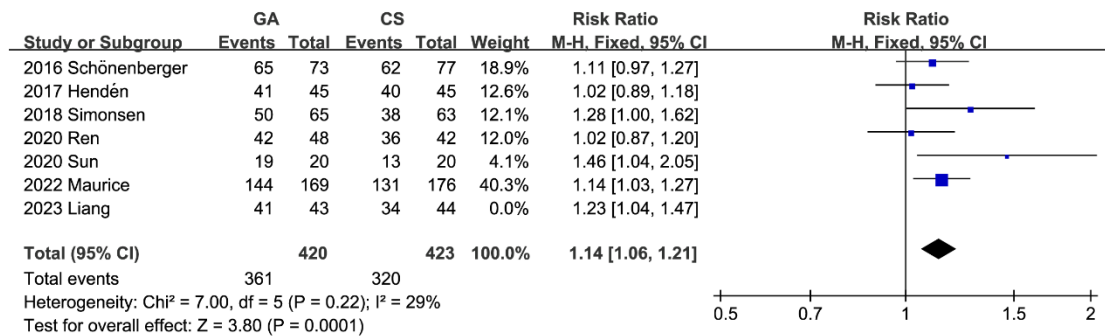

### C.Pneumonia

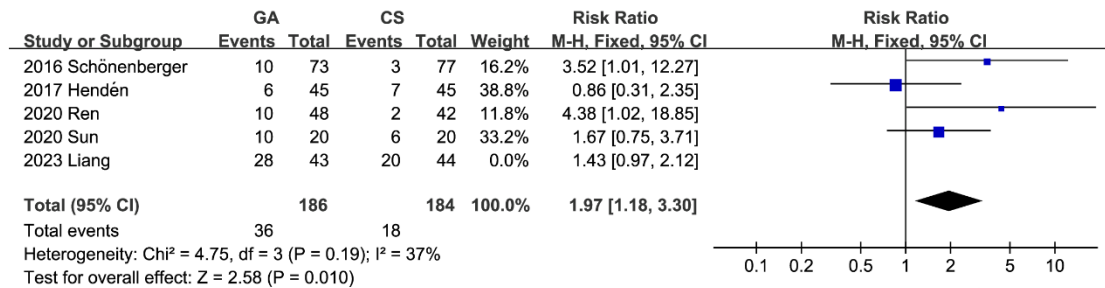

### D.mortality at 3 months

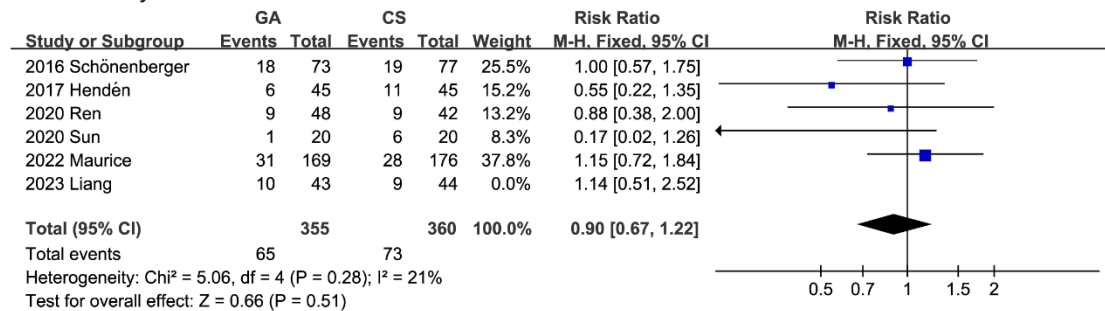

Supplement: Supplementary Figure S1 — Sensitivity analysis. [file Data_Sheet_1.pdf]
